# The Association between Body Weight Misclassification in Adolescence and Body Fat and Waist Circumference in Adulthood: A Longitudinal Study

**DOI:** 10.3390/nu14224765

**Published:** 2022-11-11

**Authors:** Abdulaziz D. Aloufi, Jake M. Najman, Abdullah A. Mamun

**Affiliations:** 1 Ministry of Health, Medina 42351, Saudi Arabia; 2 School of Public Health, The University of Queensland, Herston, QLD 4006, Australia; 3 School of Social Science, The University of Queensland, St. Lucia, QLD 4072, Australia; 4 Poche Centre for Indigenous Health, The University of Queensland, Toowong, QLD 4066, Australia

**Keywords:** body weight, weight misclassification, body fat, waist circumference, adolescence

## Abstract

This study examined the longitudinal association between adolescent body weight misclassifications and body fat and waist circumference during adulthood. A sample was derived from a large Australian birth cohort study. The data analyses were restricted to 1002 participants for whom data on both measured and perceived weight at a 14-year follow-up and the actual measure of adult body fat and waist circumference at a 30-year follow-up were available. To determine misclassifications, we compared the perceived weight with the measured weight. The results were presented as means and mean differences (with a 95% confidence interval) of the body fat percentages and waist circumference levels across the weight misclassification groups, adjusting for potential covariates. For both male and female adolescents, weight underestimation was significantly associated with an increase in body fat percentages and waist circumference in adulthood as compared to those who correctly estimated their weight. In the mean difference analyses, adolescent males and females who underestimated their weight were found to have significantly higher body fat, and waist circumference means than those who correctly estimated their weight in the unadjusted and adjusted comparisons. The adolescent males who overestimated their weight had higher body fat, and waist circumference means when they reached adulthood. Increased awareness of weight misclassification and actual weight among adolescents might contribute to better control of weight gain in adulthood.

## 1. Introduction

The adolescence period is arguably a critical developmental stage where body weight concerns become more prevalent [1]. Body weight perception may constitute an important motivation for engaging in weight modification strategies [2]. Studies have consistently provided evidence that when one’s perceived weight was compared with one’s measured weight, a large proportion of adolescents misclassified their body weight [3,4,5,6]. There is also convincing evidence that body weight misclassification, which refers to an inaccurate perception of one’s measured body weight, is associated with one’s current and future body weight [3,7,8,9,10,11,12,13]. Several studies have reported that body weight misclassification is common among adolescents and is associated with body weight management [3,14,15,16,17,18,19], suggesting that weight misclassification may be an important factor for body weight development. Underestimating or overestimating one’s body weight might have an impact on decreases or increases in one’s weight [20]. Nevertheless, very little is known about the longitudinal impact of adolescent body weight misclassification on body fat in adulthood.

Several studies have investigated the cross-sectional association between body weight misclassification and body weight and found that overweight and obese people are more likely to underestimate their body weight as compared to their underweight or normal weight counterparts, while body weight overestimation is more common for those who are underweight [3,5,8,21]. Moreover, there is some evidence to suggest that adolescents who perceive that they are overweight when this is not the case may subsequently experience an increase in body weight [11,13]. Conversely, some research suggests that body weight misclassification may limit increases in body weight [10]. For example, adolescents’ underestimation of their body weight has been associated with weight-losing behaviors [10,22]. Despite the evidence from a few relevant studies, the association of body weight misclassification with subsequent weight gain has received little attention.

There are only a few longitudinal studies that have reported an association between body weight misclassification and body composition. An 11-year Norwegian study of 1196 adolescents who were followed until they reached young adulthood found that the normal-weight adolescents who overestimated their body weight subsequently gained 0.66 body mass index (BMI) units and 3.46 cm in their waist circumference (WC) as compared to normal weight adolescents who perceived their body weight correctly [12]. Another 6-year follow-up study in Houston found that adolescents who perceived themselves as overweight, regardless of their actual body weight at the baseline, were 2.5 times more likely to gain weight after six years as compared to those who did not perceive themselves as overweight, adjusting for depression, physical activity, and dieting behaviors [13]. Additionally, longitudinal studies of adult samples in the US and UK have found that perceiving oneself as overweight led to weight gains among normal and overweight participants (0.3–0.8 BMI points from baseline to follow-up) [9]. These studies were focused mainly on measuring BMI rather than body fat and waist circumference, which are important indicators of health risks rather than BMI alone. Additionally, none of these studies considered the effect of the longitudinal association of adolescent body weight underestimation and overestimation with body fat in adulthood. Thus, investigating the impact of body weight misclassification on weight gain, especially during the adolescent period, may contribute to efforts to control excess weight gain in adulthood.

Although weight misclassification has been reported to be associated with body weight, it is unclear if misclassification may lead to a gain or decrease in body weight or weight-control behaviors. Moreover, there are several factors that may have significant associations with weight misclassification, such as racial/ethnic disparities [23], depression [24], unhealthy weight-control practices [3], physical activity [25], parental body weight status, and education level [6]. Thus, understanding the association between weight misclassification and its related factors, particularly during the early stages of one’s life course, may contribute to a better understanding of further physical and psychological health outcomes during adulthood.

While it is established that body weight misclassification affects a significant proportion of adolescents, very little is known about the longitudinal impact of adolescent weight misclassification on increases in body weight (i.e., body fat and waist circumference) in adulthood, taking into account associated predictors of weight misclassification and body weight. We hypothesize that adolescents who misclassify their body weight, particularly those who underestimate their body weight, will experience greater body fat as compared to adolescents who correctly classify their body weight. Using data from a large longitudinal Australian study, we aimed to examine the longitudinal association between adolescent body weight misclassification and body fat and waist circumference during young adulthood. The findings of this longitudinal study may guide policymakers and health professionals to develop a strategy to reduce weight misclassification in adolescence and childhood so as to prevent excess weight gain in adulthood.

## 2. Methods

### 2.1. Participants

The data were from the Mater-University of Queensland Study of Pregnancy (MUSP) and its outcomes, which was a prospective study involving mothers and their offspring. Sequential offspring who were delivered as a live singleton child and who were not adopted out before leaving the Mater Misericordiae Hospital in Brisbane, Australia, between 1981 and 1983 were selected for this study. The cohort consisted of 7223 children who were followed up prospectively for 30 years. The mothers provided details of themselves and their children at child delivery; at 3–5 days post-delivery; and at 6-month, 5-year, and 14-year follow-ups. After this, the children responded to their own questionnaires at 21-year and 30-year follow-ups. More details of this cohort study have appeared elsewhere [26,27].

**Figure 1:**
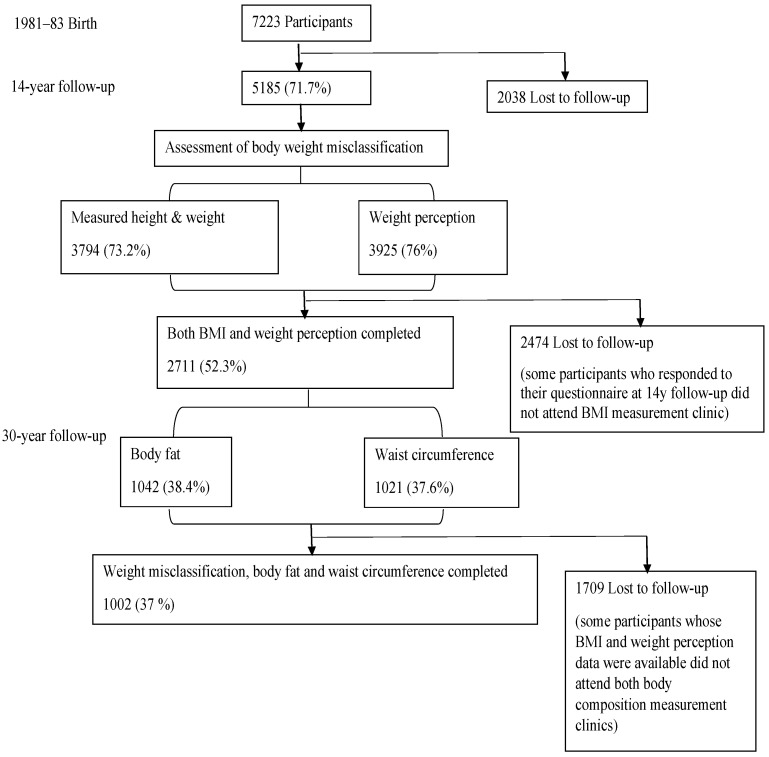
Flowchart of the MUSP cohort, body mass index, weight perception, and the analytical sample.

The analytical sample was limited to 1002 offspring for whom their perceived and measured body weight data at the 14-year follow-up and body fat and WC data at the 30-year follow-up were available (Figure 1). Those who were lost to follow-up were more likely to be from Asian or Aboriginal/Islander backgrounds; from a low socioeconomic status background; or those with a mother who was less educated, consumed tobacco or alcohol during pregnancy, or had poor mental health [26,28].

### 2.2. Measurements

Outcome (at 30-year follow-up)

Body fat and waist circumference were measured by trained research staff. Body fat was measured using the Tanita body composition monitor, which uses validated bioelectric impedance analysis technology [29]. WC was measured horizontally using a non-elastic disposable tape measure roughly in line with the umbilicus level of the participant and above the iliac crest. The tape measure was in a straight position parallel to the floor and held against the subject’s abdominal skin without compressing the skin. Two or more measures were taken at the end of the expiration phase. The averages of these measures were calculated and recorded [30,31]. 

Exposure (at 14-year follow-up) 

The participant’s weight was recorded by taking the average of two measures with a 5-min interval on a scale accurate to 0.2 kg, with the participants in light clothes. Height was measured for each participant without shoes using a portable stadiometer. To categorize overweight and obesity, Cole et al.’s international survey standard definition for overweight and obesity was used to define the BMI cut-off values for the overweight and obese categories [32]. To categorize underweight and normal weight, participants whose BMI fell under the 10th percentile were classified as underweight; the others were classified as having normal weight.

Weight perception was obtained from the adolescents’ questionnaires [26]. The adolescents were asked: “do you think of yourself as” with the following response options: “very underweight,” “underweight” “about the right weight,” “overweight,” or “very overweight.” The very underweight was merged into the underweight category, and the very overweight was merged into the overweight category. A new variable (BMI-perceived weight) was created using these merged categories (of underweight and overweight).

Weight misclassification was defined by comparing the weight perception categories with the BMI-measured weight categories. The participants who correctly estimated their perceived body weight were categorized as the “correct estimation” group. The underestimation group contained participants whose weight perception was less than their BMI-measured weight category, while the overestimation group contained participants whose weight perception was recorded as higher than their BMI-measured weight category. Weight misclassification was categorized into three levels: correct, underestimation, and overestimation.

Confounders (from the first phase to the 14-year follow-up)

The main confounders were selected based on published studies or previous knowledge regarding their association with weight misclassification or body composition [3,5,13,14,15,33,34]. These factors were listed according to the phases of this study.

For the first phase of this study: Maternal information, including maternal height and weight, maternal education and age, and the ethnicity of the parents, was obtained from the first clinical visit and maternal obstetrician records. Maternal BMI cut-off values were defined according to the standard definition of BMI [35]. Maternal education was classified according to the mother’s completion of high school (“incomplete high school,” “complete high school,” and “post-high school”), and parental ethnic origin was classified as “White,” “Asian,” and “Aboriginal Islander.” 

At the 5-year follow-up, the children’s weight and height were measured in a manner similar to that of the 14-year follow-up BMI measurement methods. The average of two measures was taken when the participants were lightly clothed, on a scale accurate to the nearest 0.2 kg, to measure their body weight, while height was measured using a portable stadiometer. Additionally, BMI cut-off values were identified based on Cole et al.’s international survey definition of overweight and obesity, while participants who were under 10% of the BMI percentile were classified in the underweight category. Too few subjects remained in the obese and underweight categories; thus, they were merged into the overweight and normal categories, respectively.

At the 14-year follow-up, several measures were obtained from the adolescents’ questionnaires. These factors included “how often do you go on a diet to lose weight?” with the following response options: “always or often” and “rarely or never”; “how many days did you do leisure physical exercise?” with the following response options: “0–1”, “2–3”, “4–5”, and “6–7 days”; and “time spent on watching television a week,” with the following response options: “less than 3 h” and “more than 3 h”. Additionally, pubertal development was divided into five stages according to the Tanner Scale [36,37]. For meaningful analysis, the first two stages were merged into one category, as were the last three stages. Additionally, mental health factors, which include internalizing behaviors (depression and anxiety) and externalizing behaviors (delinquent and aggressive behaviors), were identified using Achenbach’s Youth Self Report (YSR) [38]. The adolescents’ responses to their behavioral and psychological questionnaire items were summed up using the YSR scale, and the subjects who remained in the top 90th percentile of the scores were considered a “case.” 

### 2.3. Statistical Analysis

Regarding the analyses of missing values, the participants were divided into two groups as follows: those who were not lost at the baseline (14-year follow-up) and follow-up (30-year follow-up) periods; and those who were not lost only at the baseline period. We calculated the mean difference in body fat percentages and WC among these groups. We used the Chi-square test (χ^2^) and a *p*-value of <0.05 to perform bivariate analyses of these groups with confounders. 

The BMI-perceived weight categories were matched with the BMI-measured weight categories to determine the occurrence of weight misclassifications. The mean and standard deviation values of the body fat percentages and WC among weight misclassification categories were calculated. The statistical significance of the results was reported using a two-tailed *p*-value where a value <0.05 was used to determine the significance level. 

A multiple linear regression model was used to calculate the differences in mean and significance between the exposure (weight misclassification as an independent variable) and the outcome (body fat percentages and WC as dependent variables). Confidence intervals were used to guide the assessment of statistical significance, with the “correct estimation” group being used as the reference group. This analysis was then repeated after we included potential confounders. In this adjusted analysis, BMI at the 5-year follow-up and maternal BMI were initially involved in the model, and the rest of the factors were included in the final model. The missing values of the confounders were treated using multiple imputations. Each potential confounder was imputed before including it in the analysis. 

SAS version 9.4 (SAS Institute Inc., Cary, NC, USA) was used to perform all the calculations. 

## 3. Results

There were a total of 1620 participants for whom WC and body fat percentage at the 30-year follow-up were available. From these, the final sample comprised 1002 participants (43% male) in which the BMI-perceived and BMI-measured weight were also available at the 14-year follow-up. We compared the characteristics of the participants who were not lost to follow-up at the 14-year follow-up against those who remained at both the 14-year and 30-year follow-ups. None of the comparisons were statistically significant (see Supplementary Tables S1 and S2). 

Table 1 shows the mean and standard deviation values of body fat percentages and WC of both genders at the 30-year follow-up, stratified by weight misclassification at the 14-year follow-up. The mean values of body fat percentages and WC among males and females appeared to be higher in the underestimation group as compared to the correct estimation group. In the underestimation group, the mean of the body fat percentages of the males was 24.1% (SD: 8.5), which was higher than the mean of 20.3% (SD: 7.1) in the correct estimation group, while the mean of the WC of the males was 98.5 cm in the underestimation group (SD: 16.6), which was higher than the mean of 92.9 cm (SD: 12.3) in the correct estimation group. Similarly, among the females who underestimated their body weight, the mean body fat percentage was 37.7% (SD: 8.9), which was higher than the mean of 34.4% (SD: 8.7) in the correct estimation group, while the mean of the WC was 92.1 cm (SD: 17), which was higher than the mean of 85.9 cm (SD: 14.8) in the correct estimation group. The previous differences in the mean values of the body fat percentages and WC among those who underestimated their body weight as compared to those who correctly estimated their body weight was statistically significant (Table 2). In the overestimation analyses, while the mean of the body fat percentages and WC among the males appeared to be relatively higher as compared to the correct estimation group, the mean among the females appeared to be relatively lower as compared to the correct estimation group.

Table 2 shows the association between weight misclassification at the 14-year follow-up and body fat percentages at the 30-year follow-up for the males and females. The results are presented in mean differences with a 95% confidence interval (95% CI) across the weight misclassification categories considering the correct estimation group as the reference group. Among the males, the mean difference of the body fat percentages in the underestimation group as compared to the correct estimation group was statistically significant, as they were in the overestimation group as compared to the correct estimation group in both the non-adjusted and adjusted analyses. However, the magnitude of the mean difference between the underestimation and correct estimation groups was relatively higher (3.86%; 95% CI: 2.14, 5.58) than that of the overestimation and correct estimation groups (2.59%; 95% CI: 0.61, 4.68). Among the females, the mean difference in the body fat percentage was higher (3.33%; 95% CI: 1.33, 5.34) among the underestimation group as compared to the correct estimation group. In the overestimation group, the mean difference was not significant. The respondents who underestimated their body weight at 14 years had a substantially higher level of body fat at the 30-year follow-up.

Similar to Table 2, Table 3 shows the association of the differences between the mean values of the WC across the weight misclassification categories for males and females. Similar results to those in the body fat percentage analysis appeared in the WC analyses. In both genders, the mean difference of the WC was significant among the underestimation group as compared to the correct estimation group in the adjustment comparison. However, among the overestimation group, for the females, the mean difference was not statistically significant as compared to the correct estimation group. 

## 4. Discussion 

The current study is the first to examine the longitudinal association between body weight misclassification during the adolescent period and body fat and waist circumference during adulthood. We found that both male and female adolescents who underestimated their body weight had increased body fat levels and increased waist circumference when they were adults as compared to those who correctly estimated their body weight. We also found that adolescent males who overestimated their body weight showed an increase in body fat and waist circumference as compared to those who correctly estimated their body weight, but to a lesser degree. The adjustment of potential confounders did not substantially alter these associations. The findings of this study suggest that adolescents who misclassify their body weight may experience higher body fat and a higher WC in adulthood.

Overall, the findings of this study are consistent with those of a longitudinal study that examined the association between adolescent weight misclassification and waist circumference [12].

Previous research has indicated that those who underestimate their body weight are less likely to engage in weight-losing behaviors as compared with those who correctly estimate their body weight [3,14,15,16,18,19]. Several studies have found that body weight underestimation might be an obstacle to attempts to maintain or lose weight or to maintain a healthy diet to lose weight, especially among overweight and obese individuals [14,39]. Many individuals may not accept that they are at risk of being overweight or obese. Body weight underestimation might reflect an underlying attitude or belief that influences obesity-related lifestyles. Body weight underestimation could hinder efforts to take steps toward maintaining a healthy weight or losing harmful weight. On the other hand, body weight overestimation has been reported to be associated with going on a diet to lose weight and weight-losing behaviors; however, people who overestimated their body weight have also been reported to be prone to adopting unhealthy weight-control behaviors, such as extreme diets to lose weight or taking drugs such as diet pills or laxatives [40,41]. Body weight overestimation might lead to increased body weight over time, as weight-control behaviors used by those who overestimate their weight might be ineffective and lead to weight gain in the longer term. The findings of this study suggest that, after controlling for related confounders, the adolescent males who overestimated their body weight at the 14-year follow-up had a higher waist circumference and more body fat in their adulthood as compared to those who correctly estimated their body weight.

This study has the advantage of having data available for assessing the longitudinal association between adolescent body weight misclassification and the development of body fat and WC increases during adulthood, taking into account early-life predictors. Most of the research in this area has been cross-sectional or has not involved early-life predictors. Additionally, this study used a large representative sample that covered the adolescent and early adult periods, which are significant life stages for studying obesity and body weight misclassification. The main limitations of this study are the missing values, which may have affected the magnitude of our findings of body fat percentages and WC. However, in our baseline characteristics analysis, there was no significant difference when we compared the main characteristics of those who were not lost to follow-up at the baseline of this study with those who remained at both baseline and follow-up. Additionally, the findings did not significantly change when we applied multiple imputations to the predictors. Moreover, previous studies that used MUSP data showed that missing data generally did not have a significant impact on their findings, even after applying multiple imputations and inverse probability weighting for the missing data [26,27,28,33]. 

Further research is needed to better understand the role of body weight underestimation and overestimation in body weight fluctuations over the life course, along with other related factors associated with weight misclassification. While obesity during childhood predicts obesity during adulthood [42], our previous research found that the effect of body weight misclassification may persist from the adolescent period into adulthood. Body weight misclassification has been consistently reported to be associated with healthy weight management and attempts to lose weight [3,14,15,16,18,19]. Weight misclassification, especially body weight underestimation, during childhood, might contribute to weight gain in adulthood. Furthermore, parents’ perception of their children’s weight status might have an impact on children’s body weight, as parents usually facilitate children’s access to physical activity and influence what their children eat. Parents who underestimate their children’s body weight may be less likely to help their children to engage in weight-losing programs [43,44,45,46] as compared to parents who correctly estimate their children’s body weight. Children who live with overweight or obese parents or interact with overweight or obese schoolmates have been reported to experience more body weight misclassifications as compared to those who do not [8]. Clinical and school-based BMI screening might be useful in delivering a positive message in regard to one’s body weight and might encourage steps that can be taken to maintain a healthy weight, as well as help inform parents about their children’s weight status. These factors, along with body image distortion; body weight stigma; unhealthy diets; and other related psychosocial, environmental, and cultural factors, should be considered when studying the longitudinal association between weight misclassification and body weight. There is currently little known about the impact of interventions and the health benefits of maintaining a correct body weight perception. This is a gap in the knowledge that needs to be rectified.

## 5. Conclusions

In this paper, we report a longitudinal association between body weight misclassification during the adolescent period and the development of body fat and waist circumference during adulthood. We also found that there are early-life factors that might have an impact on such an association. Overall, the adolescent males and females who underestimated their body weight had a significant increase in their body fat and waist circumference as compared to those who correctly estimated their body characteristics. While the adolescent males who overestimated their body weight experienced an increase in their body fat and WC, the adolescent females who overestimated their body weight had a non-significant decrease in both body fat and waist circumference in adulthood. There is a case here for considering the development of educational programs that encourage adolescents and young adults to assess their body weight more accurately. In so doing, we may reduce the prevalence of weight misclassification and increase correct estimations of weight among adolescents, and also reduce the risk of excess weight and body fat in adulthood. In addition, increasing awareness of one’s own body weight status needs to be taken into consideration when addressing unhealthy weight-control behaviors, which may hinder tackling excess body weight and body fat [47,48]. In all of these policy options, there is a need to balance the concerns of adolescents regarding their body image and the need to accurately assess body weight.

## Figures and Tables

**Table 1:** Distribution of males’ and females’ body fat percentages and waist circumference at 30-year follow-up by body weight misclassifications at 14-year follow-up.

Misclassification at 14-Years F/U	Body Fat % at 30-Years F/U						WC at 30-Years F/U					
	Male			Female			Male			Female		
	N	Mean	SD	N	Mean	SD	N	Mean	SD	N	Mean	SD
Correct estimation	263	20.26	7.10	315	34.38	8.74	263	92.86	12.27	315	85.91	14.83
Underestimation	104	24.12	8.50	97	37.72	8.92	104	98.51	16.58	97	92.15	16.97
Overestimation	60	22.86	6.85	163	33.52	8.63	60	96.12	11.21	163	85.10	13.31

Abbreviations: WC: waist circumference; F/U: follow-up.

**Table 2:** Mean difference and 95% confidence interval of body weight misclassification at the 14-year follow-up with body fat percentages at the 30-year follow-up.

Misclassification at 14 Years	No.	Body Fat % at 30 Years		
		Unadjusted Mean Diff (95% CI)	Adjusted Mean Diff * (95% CI)	Adjusted Mean Diff ** (95% CI)
Male				
Correct estimation	263	0	0	0
Underestimation	104	3.86 (2.14, 5.58)	3.40 (1.66, 5.15)	3.16 (1.39, 4.93)
Overestimation	60	2.59 (0.61, 4.68)	2.74 (0.71, 4.77)	2.68 (0.56, 4.80)
Female				
Correct estimation	315	0	0	0
Underestimation	97	3.33 (1.33, 5.34)	2.85 (0.80, 4.90)	2.66 (0.56, 4.76)
Overestimation	163	−0.86 (−2.51, 0.79)	−0.89 (−2.55, 0.77)	−1.13 (−2.77, 0.52)

* Adjusted for maternal body mass index; ** adjusted for maternal body mass index, maternal age, and maternal education; at 14-year follow-up: dieting, physical activity, race, puberty development, internal and external behaviors (YSR), and TV watching.

**Table 3:** Mean difference and 95% confidence interval of body weight misclassification at the 14-year follow-up with waist circumference at the 30-year follow-up.

Misclassification at 14 Years	No.	Waist Circumference at 30 Years		
		Unadjusted Mean Diff (95% CI)	Adjusted Mean Diff * (95% CI)	Adjusted Mean Diff ** (95% CI)
Male				
Correct estimation	263	0	0	0
Underestimation	104	3.86 (2.15, 5.58)	3.40 (1.66, 5.15)	3.16 (1.40, 4.93)
Overestimation	60	3.26 (−0.14, 6.66)	3.57 (0.13, 7.02)	3.89 (0.27, 7.50)
Female				
Correct estimation	315	0	0	0
Underestimation	97	6.23 (2.73, 9.74)	5.10 (1.61, 8.58)	4.35 (0.79, 7.91)
Overestimation	163	−0.82 (−3.53, 1.90)	−0.75 (−3.42, 1.93)	−0.92 (−3.55, 1.72)

* Adjusted for maternal body mass index; ** adjusted for maternal body mass index, maternal age, and maternal education; at 14-year follow-up: dieting, physical activity, race, puberty development, internal and external behaviors (YSR), and TV watching.

## Data Availability

The raw data and analyses conducted in this study are under restrictions due to the confidentiality requirements by ethical approval committee but can be available upon receiving a plausible request and approval from the ethical committee.

## Supplementary Materials

The following supporting information can be downloaded at: https://www.mdpi.com/article/10.3390/nu14224765/s1, Table S1: Mean and mean difference of baseline characteristic between participants who were available at 14-year follow up and those who remained at 14-year and 30-year follow-ups. Table S2: Comparing baseline characteristics of participants who were available at 14-year follow up and those who remained at 14-year and 30-year follow-ups.
