# Supplementary material for: The Association between Body Weight Misclassification in Adolescence and Body Fat and Waist Circumference in Adulthood: A Longitudinal Study
Source: Nutrients. 2022 Nov 11;14(22):4765. doi: 10.3390/nu14224765 (PMC9693537; doi:10.3390/nu14224765)
Supplement: Supplementary file 1 [file nutrients-14-04765-s001.zip › nutrients-1959997-supplymentary.pdf]

Table S1. Mean and mean difference of baseline characteristic between participants who were available at 14-year follow up and those who remained at 14-year and 30-year follow-ups.

CHARACTERISTICS	Available at 14y Mean (SD)	Available at 14y and 30y Mean (SD)	Mean Diff. (95% CI)
Age at 14y	13.92 (0.34)	13.93 (0.30)	0.01 (-0.34, 0.22)
BMI at 14y	20.64 (3.81)	20.69 (3.70)	0.05 (-0.13, 0.02)
Maternal BMI	21.87 (3.92)	22.0 (3.94)	0.13 (-0.12, 0.02)

Abbreviations: 14y, 14-year follow-up; 30y, 30-year follow-up; SD, standard deviation; Mean Diff, Mean difference which indicate the difference between mean of participants who were available at 14y and 30y subtracted by those who were available at 14y; 95% CI: 95% confidence interval.

Table S2. Comparing baseline characteristics of participants who were available at 14-year follow up and those who remained at 14-year and 30-year follow-ups.

CHARACTERISTICS	Available at 14y %, (n)	Available at 14y and 30y % (n)	P-value
**Gender**			
Male	51.89 (3784)	42.61 (427)	
Female	48.11 (3475)	57.39 (575)	0.16
**Race**			
White	89.29 (6259)	94.02 (912)	
Asian	4.38 (307)	3.4 (33)	0.89
Aboriginal-Islander	6.33 (444)	2.58 (25)	
**Income**			
High	79.03 (3983)	83.55 (823)	0.77
Low	20.97 (1057)	16.45 (162)	

P-value indicates the significant difference between available at 14y and available at 14y and 30y groups.
